# Plasma Epstein–Barr virus DNA as an archetypal circulating tumour DNA marker

**DOI:** 10.1002/path.5249

**Published:** 2019-02-25

**Authors:** Wai Kei Jacky Lam, Kwan Chee Allen Chan, Yuk Ming Dennis Lo

**Affiliations:** ^1^ Department of Chemical Pathology, The Chinese University of Hong Kong Prince of Wales Hospital Shatin New Territories, Hong Kong SAR; ^2^ Li Ka Shing Institute of Health Sciences The Chinese University of Hong Kong Shatin New Territories, Hong Kong SAR; ^3^ State Key Laboratory of Translational Oncology, Sir Y.K. Pao Centre for Cancer The Chinese University of Hong Kong, Shatin New Territories Hong Kong SAR

**Keywords:** liquid biopsy, circulating tumour DNA (ctDNA), nasopharyngeal carcinoma, screening, massively parallel sequencing, size‐based diagnostics

## Abstract

Analysis of circulating tumour DNA (ctDNA), as one type of ‘liquid biopsy’, has recently attracted great attention. Researchers are exploring many potential applications of liquid biopsy in many different types of cancer. In particular, it is of biological interest and clinical relevance to study the molecular characteristics of ctDNA. For such purposes, plasma Epstein–Barr virus (EBV) DNA from patients with nasopharyngeal carcinoma (NPC) would provide a good model to understand the biological properties and clinical applications of ctDNA in general. The strong association between EBV and NPC in endemic regions has made plasma EBV DNA a robust biomarker for this cancer. There are many clinical utilities of plasma EBV DNA analysis in NPC diagnostics. Its role in prognostication and surveillance of recurrence is well established. Plasma EBV DNA has also been validated for screening NPC in a recent large‐scale prospective study. Indeed, plasma EBV DNA could be regarded as an archetypal ctDNA marker. In this review, we discuss the biological properties of plasma EBV DNA from NPC samples and also the clinical applications of plasma EBV DNA analysis in the management of NPC. Of note, the recently reported size analysis of plasma EBV DNA in patients with NPC has highlighted size as an important analytical parameter of ctDNA and demonstrated clinical value in improving the diagnostic performance of an EBV DNA‐based NPC screening test. Such insights into ctDNA analysis (including size profiling) may help its full potential in cancer diagnostics for other types of cancer to be realised. © 2019 The Authors. *The Journal of Pathology* published by John Wiley & Sons Ltd on behalf of Pathological Society of Great Britain and Ireland.

## Introduction

Circulating tumour DNA (ctDNA) analysis has demonstrated great promise in cancer diagnostics 1, 2, 3, 4. The term ‘liquid biopsy’ has been used to emphasise its ability to non‐invasively obtain cancer‐associated genomic and other information 5. However, the current clinical utility of ctDNA analysis is limited by our incomplete understanding of ctDNA biology. The need to search for molecular markers (both genetic and epigenetic) has hindered ready detection of ctDNA in many cancer types. For example, in many cancer types there are no readily usable mutational hotspots where ctDNA markers could be developed 6, 7. In contrast, circulating Epstein–Barr virus (EBV) DNA in patients with the EBV‐associated cancers 8 could serve as a good model to understand the biological properties of ctDNA in general. The tumoural origin of circulating EBV DNA thus provides one type of ctDNA that could be readily detected using many different molecular strategies.

**Figure 1:**
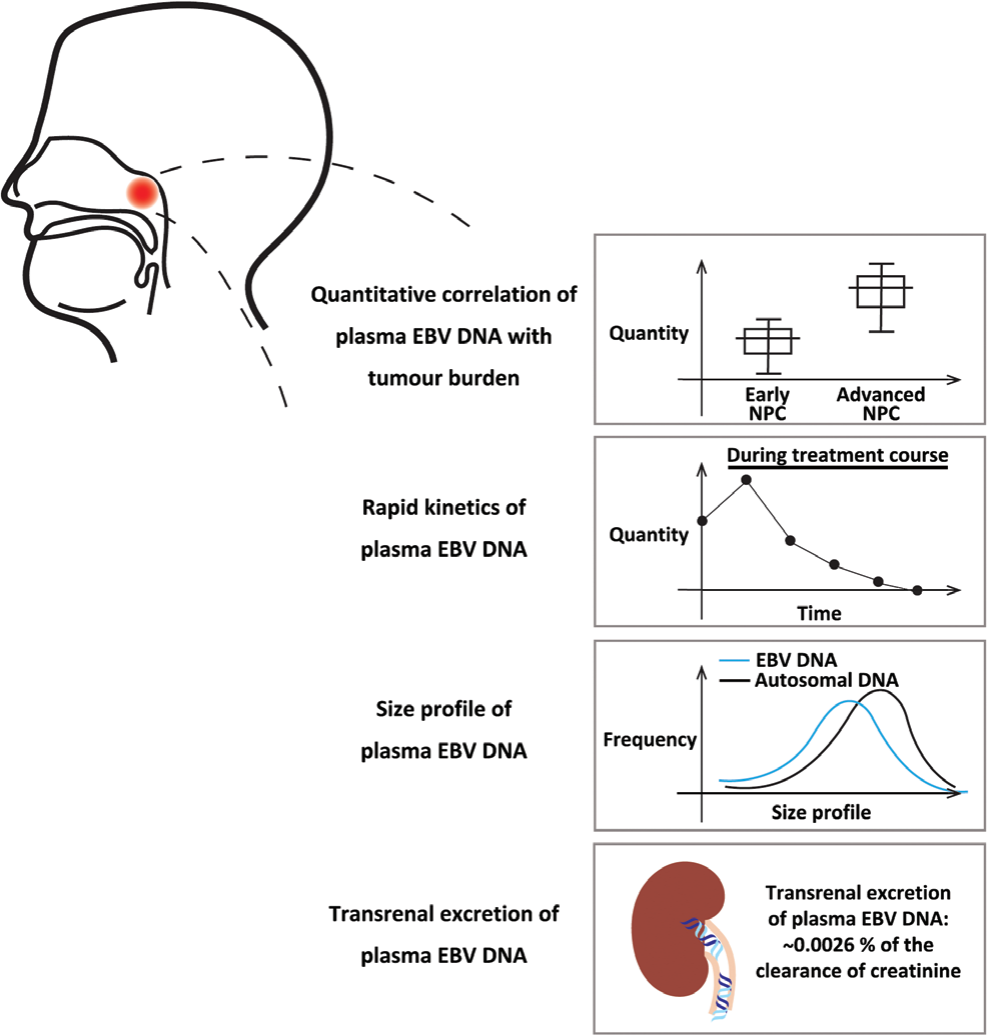
Biological properties of plasma EBV DNA in patients with NPC.

Plasma EBV DNA has been widely studied in nasopharyngeal carcinoma (NPC), as one of the EBV‐associated malignancies. NPC demonstrates distinct geographical and ethnic patterns 9 and is endemic in the southern parts of China and Southeast Asia. Virtually all NPC in endemic regions belong to the undifferentiated subtype in which virtually every cancer cell harbours the EBV genome. EBV DNA released from cancer cells into the plasma has been proven to be a robust biomarker of NPC 10. Circulating EBV DNA has been shown to be of clinical value in prognostication 11, surveillance of recurrence 12, 13 and screening 14 for NPC. In fact, plasma EBV DNA could be regarded as an archetypal example of ctDNA utility. Here, we review the molecular characteristics of plasma EBV DNA (Figure 1) and its clinical applications in NPC. The knowledge that could be gained through plasma EBV DNA analysis in NPC might illuminate the wider clinical utility of ctDNA analysis, including in cancers that do not have a viral aetiology.

## Biological properties of plasma EBV DNA in NPC patients

### Origin of plasma EBV DNA from cancer cells in patients with EBV‐associated malignancies

Our group demonstrated the presence of high concentrations of EBV DNA in the plasma of NPC patients by using real‐time qPCR 10. Plasma EBV DNA was shown to be a highly sensitive and specific biomarker for NPC. The prototype assay, which targeted the *Bam*HI‐W repeat region of the EBV genome, could detect EBV DNA in the plasma of over 95% of NPC patients and approximately 5% of the healthy controls from the study cohort. The findings were subsequently confirmed by other groups 15, 16, 17, 18, 19. EBV DNA was also shown to be present in the plasma of patients with other EBV‐associated neoplastic disorders, including Hodgkin's lymphoma, Burkitt's lymphoma, natural killer T cell lymphoma, post‐transplant lymphoproliferative disorder 20, 21 and EBV‐positive gastric carcinoma 22. Although it was estimated that 95% of the population in the world have an asymptomatic lifelong EBV infection 8, only 5% have detectable levels of EBV DNA in plasma. The virus remains latent in the B lymphocyte pool 8 with little cell turnover. Our proposed working model is that the minute amounts of EBV DNA, if any, released from cell death would not be sufficient to be detected in the circulation. This conceptual framework is built on the background of the rapid *in vivo* clearance of circulating EBV DNA. The situation changes, however, in viral reactivation, when much more EBV DNA would be released into the circulation 23. In contrast, there is a much higher cell turnover rate in cancers, e.g. up to 200 000 cancer cells/day in NPC 14, which would release sufficient cell‐free EBV DNA into the circulation to be detected.

With the discovery of circulating EBV DNA in patients with these virus‐associated cancers, researchers then asked a fundamental question about the origin of EBV DNA. Theoretically, EBV DNA in the circulation may be released from cancer cells during the process of apoptosis/necrosis 24, 25, 26, 27 or generated from viral replication. We have attempted to study the molecular characteristics of circulating EBV DNA in order to infer its origin 28. In one study, EBV DNA was measured before and after DNase I treatment of plasma samples from NPC and lymphoma patients by PCR analysis. Although plasma EBV DNA was originally present in all NPC and lymphoma patients tested, it could no longer be detected after DNase I treatment of their plasma samples. Ultracentrifugation of plasma samples showed that circulating EBV DNA from cancer patients existed in the supernatants but not in the pellet portion. As a control, a spike‐in experiment using EBV particles from the virus‐infected cell line (B95‐8) showed the opposite findings. Taken together, these findings suggest that circulating EBV DNA molecules in cancer patients exist as naked DNA fragments, which are susceptible to DNase digestion and could not be pelleted down, instead of intact virions as a result of viral replication. Lin *et al* 15 provided another strand of evidence by showing consistent genotypes of EBV DNA extracted from paired plasma and tumour samples of NPC patients.

## Positive correlation between quantitative level of plasma EBV DNA and tumour burden

One desirable characteristic of an ideal cancer biomarker is the ability to reflect the tumour burden. It has been well demonstrated that the pretreatment quantitative level of plasma EBV DNA correlated with the NPC tumour stage using qPCR analysis 10, 17, 29, 30. Patients with advanced‐stage NPC had higher pretreatment plasma EBV DNA concentrations than those with early‐stage diseases. In addition to the correlation with tumour stage, plasma EBV DNA level also demonstrated a positive linear relationship with the total tumour (both the primary tumour and regional nodes) volume quantified by volumetric analysis on MRI 31. This correlation was also illustrated in a mouse model with a positive linear relationship between plasma EBV DNA concentration and the weight of NPC tumour xenografts 32. All of these findings support a positive quantitative relationship between plasma EBV DNA level and tumour load. These results formed the foundation for clinical studies to investigate plasma EBV DNA as a marker for prognostication and surveillance of recurrence in NPC, which will be discussed below.

## Rapid kinetics of plasma EBV DNA

There are two main factors that govern the levels of EBV DNA in the circulation, namely the release of viral DNA from cancer cells and the *in vivo* clearance of EBV DNA. The release of EBV DNA into the circulation is in turn determined by the cancer cell population and also the cell turnover rate. To study the *in vivo* clearance dynamics, serial analysis of plasma EBV DNA levels in NPC patients during and after the surgical treatment procedure provides a good model for analysis, as curative surgical treatment is generally performed with an intention to eradicate all tumour cells within a short period of time (in terms of hours). With such a study design involving surgical candidates with locoregional recurrent diseases, we have shown that plasma EBV DNA was cleared at a rate that followed the first‐order kinetics model of decay; the median half‐life was 139 min 33. This number is in the same order as the half‐life of fetal DNA clearance in maternal plasma reported in the delivery model 34. Given the rapid elimination of EBV DNA in the circulation, measuring plasma EBV DNA concentrations thus provides an almost real‐time readout of tumour burden and would also be useful for monitoring recurrence.

## Size profile of plasma EBV DNA

We have previously analysed the sizes of plasma EBV DNA molecules in NPC and lymphoma patients 28. In that study, we used multiple PCR assays with different amplicon sizes to show that the majority of plasma EBV DNA from NPC patients were short DNA fragments (shorter than 181 bp). This is in concordance with the previous findings that ctDNA exists as fragmented DNA molecules 24. The fragmentation process of circulating DNA in general is non‐random and governs its size profiles. Through the use of next‐generation paired‐end sequencing, the size of each sequenced DNA molecule can be deduced by the start and end coordinates of a sequence read. Thus, it allows a high‐resolution size profiling of plasma DNA down to a single nucleotide level. The size profile of plasma DNA is one biological parameter that has been increasingly studied in a number of physiological and pathological conditions. In a pregnancy model, we have previously demonstrated that maternally derived DNA and fetally derived DNA in maternal circulation exhibit different size profiles 35. Maternal DNA peaks at a size of 166 bp, which has been postulated to represent the nucleosomal core and 10‐bp linkers at both ends. Fetal DNA, in contrast, peaks at a size of 143 bp, which corresponds to the size of the nucleosome core only. Such difference has been exploited to develop a size‐based diagnostic approach for the detection of fetal chromosomal aneuploidy in non‐invasive prenatal testing 36. Similarly, in a bone marrow transplantation model, it was shown that non‐haematopoietically derived DNA (with recipient‐specific SNP) is shorter than haematopoietically derived DNA (with donor‐specific SNP) 37. All of these findings suggest that plasma DNA molecules of different origins have different size profiles.

**Figure 2:**
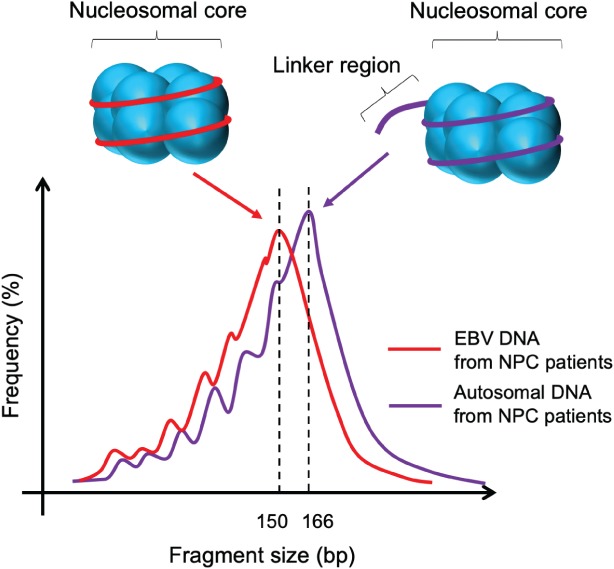
Schematic illustration of the size profiles of plasma EBV DNA and plasma autosomal DNA in patients with NPC. Both plasma EBV DNA and plasma autosomal DNA exhibit a nucleosomal size pattern. The size profile of plasma EBV DNA is on the left side of that of plasma autosomal DNA, indicating that the size distribution of plasma EBV DNA is shorter than that of plasma autosomal DNA. The difference in size may be attributed to the proportion of plasma DNA molecules with or without the linker sequence.

Recently, we performed size profiling of plasma EBV DNA from NPC patients using a sequencing‐based analysis 38. Plasma EBV DNA from NPC patients was shown to exhibit a nucleosomal size pattern, with a peak size at around 150 bp. It was shorter than plasma autosomal DNA, which was predominantly non‐tumour‐derived DNA. These results might suggest that ctDNA in general is shorter than non‐tumour‐derived DNA (Figure 2). We have also studied the size characteristics of ctDNA in patients with hepatocellular carcinoma 39. The model of hepatocellular carcinoma was chosen because copy number aberrations are frequently found in this cancer. By comparing the plasma DNA from amplified regions (enriched with ctDNA) and deleted regions (enriched with non‐tumour‐derived DNA), we reached the same conclusion as for plasma EBV DNA in NPC, that the ctDNA is in general shorter than non‐tumour‐derived DNA. The finding was also confirmed in mouse models with different types of human tumour xenograft (hepatocellular carcinoma and glioblastoma multiforme) 40. This observation underscores the importance of preserving short plasma DNA molecules during the laboratory procedures of sample preparation for ctDNA analysis. In a more specific example, the size analysis was used to improve the diagnostic performance of a plasma EBV DNA‐based NPC screening test 38, which will be elaborated below.

## Transrenal excretion

Simultaneous analysis of EBV DNA in the plasma and urine of NPC patients allows the transrenal excretion of plasma DNA in general to be explored [41]. Previously it was not clearly known how much transrenal excretion contributes to the *in vivo* elimination of circulating DNA. There were inconclusive results from studying the concentration of Y‐chromosome fragments in the urine of pregnant women carrying male fetuses 42, 43, 44, perhaps due to the low concentrations of Y‐chromosome DNA molecules in the maternal plasma. In studies involving patients with urological cancers, the tumour‐derived DNA in their urine is from both direct release into the urinary system and transrenal excretion 45, 46. In contrast, circulating EBV DNA is exclusively released from the tumour cells in NPC patients. Any EBV DNA detected in the urine samples of NPC patients is theoretically derived through transrenal excretion only. Our group has analysed the concentrations of EBV DNA in the plasma and urine of 74 NPC patients using two real‐time PCR assays with shorter (59 bp) and longer (76 bp) amplicon sizes 41. All patients (99%) except one had detectable EBV DNA in their plasma samples using the shorter‐amplicon assay. Urinary EBV DNA was detectable in 56% of patients using the PCR assay with the shorter amplicon size and 28% using the assay with the longer amplicon size. The group of patients with detectable urinary EBV DNA had a significantly higher median concentration of plasma EBV DNA than the group with undetectable urinary EBV DNA. It was calculated that only a small fraction of plasma EBV DNA was excreted transrenally, with only 0.0026% of the renal clearance of creatinine. The very low transrenal excretion of ctDNA (or circulating DNA in general) could potentially be explained by the anatomy of kidney glomeruli (pore size), the possible binding with other circulating proteins (e.g. nucleosome) and also the negatively charged nature of DNA 47, 48. The pore size of the glomerular barrier is about 30 Å 49, which is smaller than the nucleosome–DNA complex 47. The glomerular basement membrane is negatively charged, which may expel the also negatively charged DNA molecules. These findings of low transrenal excretion might be generalised to other circulating DNA.

## Clinical applications in NPC management

### For prognostication

The quantitative correlation between plasma EBV DNA concentrations and tumour burden suggests its potential as a prognostic marker. Given the rapid *in vivo* elimination of plasma EBV DNA in the circulation and therefore the almost real‐time reflection of tumour load, researchers have explored the prognostic implications of plasma EBV DNA concentrations measured at different timepoints with reference to the standard fractionated radiotherapy regime. Pre‐, mid‐ and post‐treatment plasma EBV DNA levels have been investigated.

Although pretreatment plasma EBV DNA concentration correlates with the stage of NPC, it was shown that both pretreatment level and cancer stage were independent prognostic factors for overall survival on multivariate analyses 11, 15, 19, 50. Our group demonstrated that pretreatment plasma EBV DNA level predicted the risk of local recurrence and distant metastasis within 1 year 11. The prognostic effect has been separately analysed in patients with early‐ and advanced‐stage NPC. Among patients with advanced‐stage NPC (stage III and IV), Lin *et al* 15 reported an inferior overall survival and relapse‐free survival for patients with a higher pretreatment plasma EBV DNA concentration (with a cut‐off at 1500 copies/ml). For early‐stage NPC patients (stage I and II), we have shown that high pretreatment levels (at a cut‐off of 4000 copies/ml) strongly predicted unfavourable survival (overall, disease‐specific and distant metastasis‐free survival) in the long‐term follow‐up 50. Early‐stage (stage I or II) cancer patients with high EBV DNA levels had an overall survival similar to that of stage III disease, whereas those with low levels had survival similar to that of stage I disease 50. These findings suggest that the pretreatment EBV DNA level provides additional prognostic information and might reveal tumour biology and aggressiveness independent of the tumour stage. It has been proposed to incorporate the quantitative level of pretreatment plasma EBV DNA into the current staging system of NPC 51. As the first step, there is ongoing international collaborative effort to harmonise the PCR‐based assay for quantitation of EBV DNA 52.

The standard treatment for primary NPC is a fractionated course of radiotherapy. Serial analysis of plasma EBV DNA during the course of radiotherapy could reflect the change in the tumour cell population and might therefore imply the treatment response. As a proof‐of‐concept study 53, we measured the plasma EBV DNA concentrations of primary NPC patients serially during radiotherapy. There was an initial rise in the concentration of EBV DNA during the first treatment week, which was postulated to be due to its release by treatment‐associated cancer cell death. The rise was followed by a gradual drop in concentration over the treatment course. The median half‐life among the NPC patients studied was 3.8 days. To further explore the potential, we prospectively analysed the plasma EBV DNA concentrations at the midpoint of the treatment course (4 weeks after initiation of radiotherapy) for 107 NPC patients receiving radiotherapy/chemoradiotherapy 54. The study was based on the postulate that patients with a faster plasma EBV DNA clearance and therefore undetectable mid‐treatment level would have a more radiosensitive tumour. Mid‐treatment plasma EBV DNA was shown to be the only independent prognostic factor (not even pretreatment EBV DNA) for distant failure, disease‐free survival and overall survival. Patients with detectable mid‐treatment EBV DNA represented an at‐risk group with an unfavourable response to treatment. It suggests the prognostic value of studying the *in vivo* dynamic of plasma EBV DNA and ctDNA in general 55. The observation could pave the way for future interventional studies with either escalation or de‐escalation of treatment regime based on the plasma EBV DNA molecular response.

Post‐treatment analysis of plasma EBV DNA aimed to reflect the residual tumour load after completion of treatment 56, 57, 58. In our study involving 170 NPC patients of different stages, post‐treatment EBV DNA level (at 1 month after the completion of treatment) was shown to allow the prediction of recurrence and progression‐free survival 56. Lin *et al* 15 showed that NPC patients with any detectable levels of post‐treatment plasma EBV DNA (at 1 week after the completion of radiotherapy) had a worse overall and relapse‐free survival. As post‐treatment plasma EBV DNA level predicts the risk of future relapse, Chan *et al* 59 conducted a prospective, multicentre, randomised controlled trial to evaluate the use of adjuvant chemotherapy in the high‐risk group of patients with a detectable level of post‐treatment plasma EBV DNA (at 6–8 weeks after the completion of radiotherapy). The post‐treatment level was again shown to predict worse prognosis. However, the use of adjuvant chemotherapy did not improve the relapse‐free survival among patients with detectable post‐treatment plasma EBV DNA. The authors postulated that the lack of response may be attributed to the use of the same chemotherapeutic agent (cisplatin) in the adjuvant setting as in the primary treatment regimen. Patients with detectable post‐treatment levels might already represent a group with treatment resistance and therefore no benefits of adjuvant chemotherapy were observed.

Similarly, for EBV‐associated haematological malignancies, plasma EBV DNA was also shown to be a significant prognostic marker. Positive mid‐ or post‐treatment EBV DNA levels predict inferior survival in Hodgkin's lymphoma and natural killer T cell lymphoma 20, 60, 61, 62.

The ability to prognosticate by pre‐, mid‐ and post‐treatment plasma EBV DNA levels actually reflects the potential of quantifying ctDNA at different treatment timepoints for other types of cancer. This concept might also apply to ctDNA analysis for other types of cancer. To illustrate the concept, we demonstrated the clearance of tumour‐associated copy number aberrations in the postoperative plasma samples of four patients with hepatocellular carcinoma receiving surgical treatment 63. Tie *et al* 64 analysed the postoperative ctDNA levels in patients with stage II colon cancer by targeted sequencing. Postoperative ctDNA status was found to independently predict the risk of recurrence, in addition to T stage, on multivariate analysis. These findings echo those of plasma EBV DNA studies that quantitative analysis of ctDNA could provide additional information for risk stratification.

### For surveillance of recurrence

Another main clinical application of plasma EBV DNA analysis in the management of NPC is for surveillance of recurrence 12, 13, 65, 66, 67. The level is expected to drop to an undetectable level after curative treatment and tumour eradication. It is intuitive that any rise in the plasma EBV DNA concentrations in the subsequent follow‐up period could potentially signify a disease relapse. In fact, it has been suggested that the rise could precede clinical symptoms by weeks to months 68. Currently, regular plasma EBV DNA measurement every 3–6 months is used as an adjunct to endoscopy and imaging in the clinical surveillance protocol of treated NPC patients. Similarly, in other types of cancer, Tie *et al* 64 demonstrated the utility of ctDNA analysis for monitoring of recurrence among colon cancer patients. Concurrent ctDNA and carcinoembryonic antigen analyses showed a higher sensitivity for the detection of recurrence.

### For screening

To utilise plasma EBV DNA analysis for NPC screening, there is one major concern over the sensitivity for the detection of early/presymptomatic stage of NPC with a low concentration of plasma EBV DNA. Previous studies have reported lower sensitivities for the detection of stage I or II NPC compared with advanced‐stage disease using PCR‐based assays 13, 17, 30. To evaluate its use for screening, we conducted a large‐scale prospective study in Hong Kong, which is an endemic region 14. More than 20 000 middle‐aged men who did not have any symptoms of NPC were recruited. All recruited subjects were tested for plasma EBV DNA using qPCR analysis. Subjects who had detectable plasma EBV DNA on two consecutive tests were defined as ‘screen‐positive’ and would undergo confirmatory investigations, including nasoendoscopy and MRI. Thirty‐four subjects were identified to have NPC by the screening protocol. One subject out of 19 865 screen‐negative subjects was found to have stage II NPC within 1 year after the test. The screening protocol thus achieved a high sensitivity of 97.1%. Importantly, among the 34 NPC patients identified by screening, 24 (70%) had early‐stage diseases (stages I and II) and the remaining 10 patients had advanced‐stage diseases (stages III and IV). In contrast, only approximately 30% of NPC patients would present symptomatically at an early stage and 70% at an advanced stage without screening, according to the local cancer registry 69. The earlier detection was accompanied by more superior progression‐free survival among the NPC patients identified by screening. These findings validated the use of plasma EBV DNA analysis for screening NPC and eliminated the concern of inadequate sensitivity for such a purpose.

ctDNA has been actively pursued as a non‐invasive screening tool for other different types of cancer 70. Again, one major concern has been the sensitivity for identifying early‐stage cancers 71, 72. It has been demonstrated that there were lower concentrations of ctDNA in early‐stage cancers by detecting genetic mutations in plasma DNA 73, similar to the observations of plasma EBV DNA in NPC. In a recent case–control study involving 1005 patients of different cancer types and 812 healthy controls, Cohen *et al* 74 used amplicon‐based sequencing for the detection of cancer‐associated mutations in 16 genes. They reported sensitivities of 43 and 73% for stage I and II cancers, respectively. These results highlight the current technical challenge of ctDNA analysis used for cancer screening purpose. In fact, the qPCR‐based analysis of plasma EBV DNA for screening NPC 14 might shine light on the sensitivity issue of ctDNA‐based assays for other cancer types. The PCR assay used for screening NPC targets the *Bam*HI‐W repeat region of the EBV genome, with approximately 10 repeats per genome. Assuming that there are some 50 viral genomes in each NPC cell, there are thus of the order of 500 molecular targets per cancer genome that are analysed by the PCR assay. A similarly high level of sensitivity as in the detection of early NPC might be feasible if we could achieve such a number of targets in the ctDNA‐based assay.

Approximately 5% of the general population harbour EBV DNA in their plasma 10, 75. In the NPC screening context, these subjects contribute to the false‐positive group and would need to undergo unnecessary confirmatory procedures. However, they have indistinguishable levels of plasma EBV DNA from early‐stage NPC patients by PCR‐based quantitation 38. In the prospective screening study 14, we adopted a two timepoint testing protocol of plasma EBV DNA analysis. Participants who had detectable EBV DNA in their baseline plasma samples would be retested again 4 weeks after the first test. Those who also had detectable EBV DNA in the follow‐up test would be defined as ‘screen‐positive’. This is on the basis that NPC patients should have continuous release of EBV DNA from the cancer cells into the circulation, whereas non‐NPC subjects tend to have transiently positive results 76. This strategy was shown to substantially reduce the false‐positive rate from 5.4 to 1.4%, with a positive predictive value of 11.0% in our target population. However, it will be logistically inconvenient to launch a mass screening programme based on this two timepoint protocol.

In our 20 000 subject screening cohort, we noticed that participants of older age were more likely to have detectable plasma EBV DNA while not having NPC 23. Another interesting observation was that there was a higher proportion of participants having detectable EBV DNA but no NPC on the days of blood collection with lower ambient temperatures 23. All of these findings hinted at the presence of an immunocompromised state and viral reactivation in those non‐NPC subjects with plasma EBV DNA positivity. Given the different origins of EBV DNA between NPC and non‐NPC subjects, our group therefore postulated the presence of different molecular characteristics between the two groups 38. In results from sequencing‐based analysis, NPC patients were shown to have a higher proportion of EBV DNA reads in their plasma samples than non‐NPC subjects (quantitative‐based analysis). Second, NPC patients had a different size profile of plasma EBV DNA from non‐NPC subjects (size‐based analysis). In detail, plasma EBV DNA from NPC patients exhibited a typical nucleosomal size profile with a peak size of around 150–160 bp, whereas EBV DNA from non‐NPC subjects did not. Based on the quantitative and size differences of plasma EBV DNA, we developed a second‐generation sequencing‐based screening test that could achieve a modelled positive predictive value of 19.6% in our test population 38. This value is almost double that obtained from the two timepoint PCR‐based protocol. Of note, this second‐generation test requires only a single timepoint testing. Our study result has illustrated that size profiling of plasma DNA could bring another dimension to the current approach for ctDNA analysis. This has also highlighted the importance of understanding the biology and molecular characteristics of plasma DNA in general, which could be translated into improvements in the diagnostic performance of ctDNA analysis.

## Conclusion

Since plasma EBV DNA was recognised as a biomarker of NPC researchers have been working extensively to validate various clinical applications in prognostication, surveillance of recurrence and screening. As a result, plasma EBV DNA is one of the most widely used ctDNA markers to date. With technological advances, especially in next‐generation sequencing, ctDNA analysis demonstrates great potential in cancer diagnostics for different cancer types. However, the current bottlenecks encountered in ctDNA‐based biomarker research have presented some challenges that would need to be solved. Researchers could gain insights from the plasma EBV DNA analysis model into solving these challenges. Furthermore, plasma EBV DNA in NPC has provided a good model to study the biology of ctDNA, when plasma EBV DNA has already been shown to share similar molecular characteristics as ctDNA. A better understanding of the molecular features of ctDNA would improve such analysis for cancer diagnostic purposes, as illustrated in the example of size profiling of plasma EBV DNA, which improves the specificity for screening NPC. We envision that ctDNA could become a powerful tool in cancer diagnostics and has the potential to revolutionise cancer management.

## Author contributions statement

All authors wrote the manuscript.
